# Exploring the Effectiveness of Technology‐Based Interventions in Aphasia Rehabilitation: A Systematic Review

**DOI:** 10.1111/1460-6984.70322

**Published:** 2026-09-08

**Authors:** Bader Alhejji, Stuart Cunningham, Samantha Dorney, Rebecca Palmer

**Affiliations:** ^1^ College of Health Rehabilitation Sciences Speech and Hearing Disorders King Abdulaziz University Jeddah Saudi Arabia; ^2^ School of Allied Health Professions Nursing and Midwifery University of Sheffield Sheffield UK; ^3^ Department of Neuroscience University of Sheffield Sheffield UK; ^4^ School of Medicine and Population Health University of Sheffield Sheffield UK

## Abstract

**Background:**

Although speech and language therapy (SLT) is central to post‐stroke aphasia rehabilitation, global SLT provision often falls short of recommended dosages. In response, interest has grown in technology‐based interventions, including therapy software, virtual reality (VR) and artificial intelligence (AI) tools. However, current evidence for the effectiveness of technology is fragmented, and no recent review offers a comprehensive synthesis across these three modalities.

**Aim:**

This review examined the range of technologies used in aphasia assessment and therapy and summarised their effectiveness across different intervention targets. The two research questions were: (1) What types of technology have been investigated for assessing and treating people with aphasia (PWA)? (2) How effective is the use of technology in the assessment and treatment of PWA?

**Methods:**

A systematic search of four databases (PubMed, PsycINFO, Web of Science and Scopus) covering the period 2013 to May 2026 identified 67 included studies, of which 14 were randomised controlled trials. Studies reporting quantitative outcomes, were peer‐reviewed, and focused on technology‐based intervention for PWA were eligible. Quality was appraised using the NICE checklist. The GRADE framework was applied to evaluate certainty of evidence for each intervention target. Findings were then synthesised narratively due to heterogeneity across study designs, and outcome measures.

**Results:**

Three technology types were identified: computerised speech and language therapy (CSLT) (38 studies), VR (17 studies) and AI (13 studies). AI was used predominantly for aphasia assessment and classification. The strongest and most consistent evidence related to word‐finding, where high certainty of evidence was supported by multiple RCTs delivering therapy at or above the recommended 20‐h threshold. For language production and comprehension, functional communication, and reading, outcomes were more variable, reflecting moderate certainty of evidence, and inconsistent dose adherence. Writing interventions received a low certainty rating, reflecting small samples, limited blinding and task‐specific rather than generalised gains. Across domains, higher‐dose studies were consistently associated with better outcomes, which may suggest that technology functions primarily as a tool to enable high‐intensity practice rather than as an independently effective treatment ingredient.

**Conclusion:**

CSLT, VR and AI tools show promise as adjuncts to face‐to‐face SLT for aphasia assessment and rehabilitation. Word‐finding interventions delivered at recommended doses have the strongest evidence base. Some studies did not use technology to support the recommended therapy dose. For other intervention targets, larger, higher‐dose trials are needed. Future research should also examine whether integrating different technology types could offer additional clinical benefit.

**WHAT THIS PAPER ADDS:**

*What is already known about the subject*

- Previous systematic reviews have demonstrated the emerging role of technology in aphasia rehabilitation, with earlier work focusing primarily on computer‐based therapy or AI technologies. However, these reviews were either narrow in scope (targeted specific technology type), or outdated.

*What this study adds to the existing knowledge*

- This review provides an updated, cross-technology synthesis encompassing AI, virtual reality, and computerised speech‐and‐language therapy. It outlines how these tools were applied within the studies in the literature. The review also identifies persistent limitations in therapy dosage across studies, underscoring the need for future higher‐dose trials to confirm the certainty of evidence across different intervention targets.

*What are the clinical implications of this study?*

- The growing evidence for computerised speech and language therapy, virtual reality, and artificial intelligence tools continues to support their role as adjuncts to face to face SLT, particularly for language assessment and targeted word‐finding interventions. These technologies may extend therapy provision beyond clinical hours, enable therapeutic doses of practice to be achieved, and improve consistency in assessment procedures.

## 1 Background

Aphasia is a language impairment that is often caused by a stroke, with around one in three stroke survivors affected (Goodglass et al. 2001). For many individuals, the condition can be long‐lasting and deeply disruptive, making it difficult to communicate and affecting their identity, relationships and day‐to‐day involvement in life (Cruice et al. 2003; Hilari and Byng 2009). It can interfere with all aspects of language, including understanding and producing spoken language, as well as reading and writing (Brady et al. 2016). Speech and language therapy (SLT) is the principal form of rehabilitation for people with aphasia (PWA), with professional guidelines emphasising the importance of early, intensive and person‐centred intervention to maximise outcomes (Royal College of Speech and Language Therapists 2020). Broadly, SLT approaches fall into two categories: those targeting language impairments directly, and those focused on improving functional communication in everyday life. Impairment‐based interventions aim to restore specific aspects of language by engaging clients in structured tasks that target elements such as phonological processing, word retrieval and sentence construction (Fridriksson and Hillis 2021). Functional communication approaches prioritise practising language in real‐life contexts and using personalised vocabulary that supports interaction and participation (Brady et al. 2020). Others have identified functional communication approaches to achieve communicative success in everyday contexts, may involve strategies such as training communication partners, adapting the environment to patient needs by adding personalised content to facilitate interaction and participation (Doedens and Meteyard 2022).

Dosage has emerged as a key factor for meaningful recovery for PWA following stroke (RELEASE Collaborators 2022). Dosage refers to the total amount of therapy delivered (in hours). Evidence from the RELEASE study shows that the most substantial benefits of therapy for PWA occur with a dose of therapy of between 20 and 50 h. Therapy sessions should occur between three and five times a week, with a total weekly dose of between 2 and 4 h. Despite these findings, many individuals worldwide receive much less therapy than is recommended. For instance, in the UK, Palmer et al. (2019) found that individuals more than 3 months’ post‐stroke received a median of just 380 min of SLT over a 3‐month period, which is equivalent to only 30 min per week. In Australia, 80% of SLTs in acute care settings reported providing therapy at low to moderate intensities, typically offering only one to two sessions weekly, amounting to around 90 min of therapy a week (Verna et al. 2009). A range of barriers contribute to this persistent shortfall in SLT dose. These include limited capacity, the high cost of long‐term rehabilitation and a lack of continuity in care after discharge from inpatient services (Arntz et al. 2023; Monnelly et al. 2023). In response to these problems, there is growing interest in technology‐based interventions that can support provision of services and increase therapy intensity and accessibility for PWA (Nichol et al. 2023).

Over the past two decades, tools such as computer programs, artificial intelligence (AI) and virtual reality (VR) have been developed to supplement or extend face‐to‐face therapy (Kurland et al. 2014; Palmer et al. 2019; Qin et al. 2020; Marshall et al. 2023). Recent clinical guidelines provide direction on rehabilitation delivery and the role of technology: The Royal College of Physicians’ National Clinical Guideline for Stroke (2023) explicitly states that PWA should be offered access to appropriate practice‐based digital therapies to overcome access barriers. Additionally, the National Institute for Health and Care Excellence (NICE) stroke guidelines (2023) set standards for stroke rehabilitation (including SLT intensity) and support the use of computer‐based therapy for word finding for PWA.

Although interest in technology‐based approaches for aphasia rehabilitation has grown steadily, current research in this area remains fragmented and uneven (Kidd and Garcia 2022). The last review that addressed a wide range of technological interventions in aphasia rehabilitation was conducted a decade ago by Zheng et al. (2016). Since then, other reviews have explored developments within this field but with a narrow focus or different aims. For example, Repetto et al. (2020) concentrated on VR and mobile apps, while Adikari et al. (2023) examined the applications of AI for aphasia diagnosis and management. Although these contributions are valuable, they highlight the need for a broader synthesis of technology use, and potential effectiveness in aphasia rehabilitation.

## 2 Methods

This review was conducted and reported in accordance with the Preferred Reporting Items for Systematic Reviews and Meta‐Analyses (PRISMA) 2020 guidelines (Page et al. 2021) **(see** **Appendix** **2**).

### 2.1 Research Questions

1. What types of technology have been investigated for assessing and treating PWA?
2. How effective is the use of technology in the assessment and treatment of PWA?

### 2.2 Scope of the Review

The scope of this systematic review was defined using the PICO framework. The **population** (P) of interest consisted of PWA. The **intervention** (I) focused on technology‐supported language assessment and treatment. The **comparison** (C) included usual care or no therapy, and the **outcome** (O) considered was the effectiveness, improvement and accuracy of the interventions.

For this review, four databases were selected: **PubMed**, **PsycINFO**, **Web of Science**, and **Scopus**. These platforms have been used in previous systematic reviews investigating the role of technology in the assessment and treatment of aphasia (e.g., Repetto et al. 2020). Research in this area is diverse, encompassing a broad range of technological tools, including computerised speech and language therapy (CSLT) interventions (e.g., Palmer et al. 2019), VR (e.g., Cherney et al. 2021) and AI (e.g., Qin et al. 2020). No grey literature search was conducted, and backward or forward citation searching was not performed. The search was restricted to peer‐reviewed publications indexed within the four selected databases.

Searches for this review were conducted in two phases. The initial search was conducted between **1 April 2024 and 5 August 2024**, covering studies published from 2013 to 2024. An updated search was conducted in May 2026, covering the period from **1 January 2024 to 18 May 2026** in order to ensure the review captured the most recently published evidence prior to publication. A 1‐year overlap between the two phases was retained to ensure no eligible studies were missed at the boundary. The following search terms were combined in the selected databases.

(Aphasi* **OR** Dysphasi* **OR** PWA **OR** Stroke survivors **OR** Stroke victims **OR** Stroke patients) **AND** (Artificial intelligence **OR** Natural language processing **OR** Virtual Reality **OR** Augmented reality **OR** Gamification **OR** Games **OR** Tech* **OR** Tablet‐ based **OR** Computer **OR** Software **OR** Mobile technology **OR** Mobile apps) **AND** (Assessment **OR** Evaluation **OR** Intervention **OR** Treatment **OR** Therapy **OR** self manage*).

Full Boolean search strings for each database, including all applied limits and filters, are provided in **Supplementary File** **1**.

### 2.3 Eligibility Criteria

1. **Date**: The review was limited to studies published between **1 January 2013 and 18 May 2026**.
2. **Language**: Only studies published in English were considered for this review.
3. **Population**: Eligible studies must include individuals with aphasia as a primary target group.
4. **Interventions**: Included studies must examine technological interventions aimed at language assessment or rehabilitation for PWA.

Studies focusing on augmentative and alternative communication (AAC), teletherapy or transcranial direct current stimulation (tDCS) were excluded. AAC was excluded due to its emphasis on providing alternative ways of communicating rather than supporting recovery of verbal language through technology‐based interventions. Teletherapy studies were excluded if the primary purpose of the technology was to facilitate therapist‐delivered sessions remotely, such as videoconferencing platforms used solely as a substitute for face‐to‐face SLT, as these represent a mode of service delivery rather than a distinct therapeutic tool. Studies were retained where technology served as the primary intervention method, including those with an element of remote or hybrid supervision, provided that the technology itself enabled independent or self‐managed practice by the participant. This distinction reflects the review's focus on the therapeutic potential of technology as a tool to extend and intensify practice, rather than as a delivery channel for face‐to‐face therapy. Addition ally, tDCS was also excluded, as it involves direct neuromodulation rather than engaging users in interactive, task‐based therapy (Floel 2014). Moreover, tDCS requires administration by trained professionals and does not align with the idea of increasing therapy dose for PWA.

5. **Data Type**: Only studies reporting quantitative outcomes related to the effectiveness of the technological interventions were included.
6. **Study Design**: Accepted designs included experimental studies, randomised controlled trials, quasi‐experimental research, case studies, cohort studies, observational, and analytical studies and case–control studies. Editorials, letters, conference abstracts and academic theses were excluded.

Search results were screened for eligibility in two stages: first by title and then by abstract. Duplicates were removed using Excel software. Screening was conducted primarily by the first author. To ensure screening reliability, approximately 25% of the total records (*n* = 687) were independently screened by a third author at all screening stages (title, abstract and full text as needed). Only two discrepancies were identified across this subset, which were resolved through discussion between the two reviewers (agreement rate = 99.7%).

### 2.4 Ethical Considerations

The current study is a systematic review of previously published peer‐reviewed articles and did not involve primary data collection or direct contact with human participants. Therefore, formal institutional ethical approval was not required. However, the review adhered to rigorous academic and ethical reporting frameworks by conducting all data extraction, quality checks and reporting in strict alignment with the PRISMA (2020) guidelines.

### 2.5 Data Extraction Process

An Excel spreadsheet was created to extract and organise information from each included study. This spreadsheet captured key information relevant to understanding the effectiveness of technological interventions in the assessment and treatment of aphasia including: the study aim, design (e.g., randomized controlled trials), participant characteristics (e.g., sample size, aphasia type), intervention length and dose and the specific outcome measures used to evaluate success; intervention accuracy, efficacy, effectiveness; clinical application (e.g., setting of use); degree of personalisation, and presence of feedback mechanisms. Where studies reported multiple outcomes or time points, data were extracted for final post‐treatment results when available. The data extraction process also recorded whether each study had a comparison group, the specific intervention target (e.g., improvement in word finding), the type of technology used (e.g., VR), and the language used within the intervention. The clinical use of the technology, including whether it was delivered during face‐to‐face SLT sessions or used independently at home, was also documented.

If outcome reporting was inconsistent or unclear, the reviewers agreed on a set of guiding principles in advance and applied them consistently (i.e., prioritising final post‐treatment outcomes over interim results, selecting the primary outcome specified by the study where multiple outcomes were reported, and extracting data from the main participant group when subgroup data were presented). Where data were missing or unclear, no assumptions were made; fields were recorded as ‘Not reported’ (NR). The authors jointly discussed and agreed on the most relevant data items to be extracted prior to the process. Data extraction was then conducted by one reviewer (the first author).

### 2.6 Data Synthesis

All included studies were eligible for synthesis. A narrative synthesis approach was adopted to integrate findings across the included studies, following guidance by Popay et al. (2006). Meta‐analysis was not feasible due to substantial heterogeneity across the included studies. The specific sources of this heterogeneity included variation in study designs, ranging from single‐case experimental designs to multicentre RCTs; diversity in intervention targets, technology types and delivery contexts; inconsistency in outcome measures, with studies using different naming batteries, standardised language assessments and bespoke tools that are not directly comparable; and considerable variation in therapy dose and follow‐up periods. Even within the word‐finding domain, where the largest number of RCTs was concentrated, partial quantitative synthesis was not appropriate, as studies reported a mixture of between‐group and within‐group outcomes, used different primary outcome measures such as the Object and Action Naming Battery, Word Retrieval Test, and Philadelphia Naming Test, and targeted different sets of personally relevant words, making pooled effect estimates uninterpretable. No attempt was made to pool results statistically, as preliminary examination of outcome measures and reporting formats confirmed that pooled estimates would not be meaningful even within the most homogeneous subgroups.

Studies were first charted to summarise their key features and outcomes, and then iteratively compared to identify patterns, relationships and differences. Groupings were developed according to the primary intervention target rather than by technology type, because similar technologies were used for different therapeutic aims and in diverse clinical settings. Grouping by intervention target provided a clinically meaningful way to compare how technology supported specific aspects of language rehabilitation across studies. Following Popay et al. (2006), similarities and differences were explored within each intervention target by examining the dose and delivery context of interventions, and the consistency and magnitude of reported outcomes. Methodological features, including sample size, design and risk of bias, were also considered to understand how study quality contributed to convergence or divergence in findings. This process facilitated an interpretive synthesis that accounted for both outcome patterns and the contextual factors shaping them.

### 2.7 Quality Assessment of the Included Studies

A quality assessment of the included studies was conducted in two stages. The two‐stage approach was adopted because individual study quality and overall certainty of evidence address different but complementary questions. The National Institute for Health and Care Excellence (NICE) checklist was applied at the individual study level to evaluate how well each study was designed and conducted, and to identify specific sources of bias within each study. The GRADE (Grading of Recommendations, Assessment, Development and Evaluation) framework was then applied at the level of the evidence body, drawing on the NICE ratings alongside other criteria including inconsistency, indirectness, imprecision and publication bias, to determine the overall confidence that can be placed in the findings for each intervention target. This sequential approach is consistent with established systematic review methodology, in which study‐level risk of bias informs, but does not solely determine, the certainty of the wider evidence base.

First, each study was assessed using NICE quality appraisal tool, specifically the **Appendix F** 27‐question checklist. This checklist evaluates five key sections: population, methods, outcomes, analysis and summary. **Appendix** **1** sets out the NICE response options used for each intervention checklist item. Another independent researcher (the third author) reviewed approximately 20% of the included studies (*n* = 13) to verify the consistency and accuracy of the NICE quality appraisal ratings. One discrepancy was identified, which was resolved through discussion between the two reviewers, yielding an overall agreement rate of 92.3% (**see** **Table** **3**). Second, following the individual study assessments, the GRADE framework was employed to evaluate the strength and certainty of the evidence for the identified outcomes. This framework evaluates studies based on their design, with randomised controlled trials (RCTs) starting as high‐quality evidence and observational studies as low‐quality evidence. Factors that can downgrade the quality of evidence include study design limitations (risk of bias), inconsistency of results, indirectness of evidence, imprecision and publication bias (Schunemann et al. 2013), (see **Table** **4**).

## 3 Results

**FIGURE 1:**
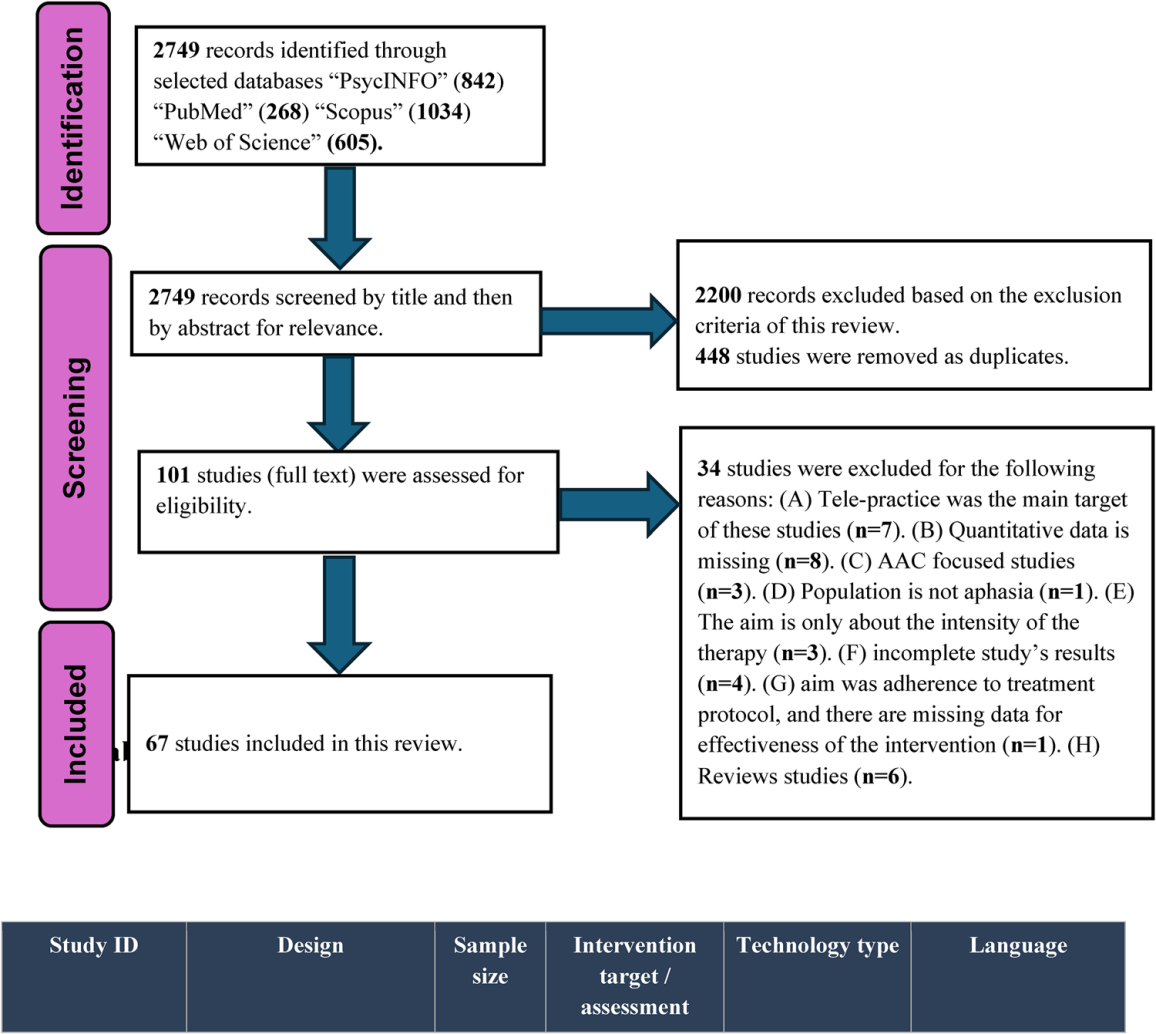
PRISMA flowchart for the study selection process.

The PRISMA flowchart (**Figure** **1**) shows the results of the study selection process.

### 3.1 Study Design

The 67 included studies include a range of study designs, sample sizes and technologies aimed at improving or assessing language abilities in PWA. Experimental studies constituted the largest group at 29.85% (***n* = 20**), typically employing non‐randomised or within‐subject pre–post designs, generally considered preliminary in nature and focusing on feasibility, usability, or early evidence of effectiveness rather than strong evidence. Randomised controlled trials (RCTs) represented 20.90% (***n* = 14**), providing the strongest level of individual study evidence in the review. Observational and analytical studies accounted for 17.91% (***n* = 12**), followed by case series at 5.97% (***n* = 4**). Quasi‐RCTs and controlled clinical trials each represented 4.48% (***n* = 3**). Case studies, single‐case experimental designs and pre‐post cohort or group analysis studies each comprised 2.99% (***n* = 2**). The remaining designs (prospective single‐group, pilot non‐inferiority, single‐case crossover, single‐case pilot and non‐concurrent multiple baseline single‐case experimental) each contributed one study (1.49%) (**as shown in** **Table** **1**).

**TABLE 1:** Study characteristics.

Study ID	Design	Sample size	Intervention target/Assessment	Technology type	Language
(Abad et al. 2013)	Observational/analytical	16	Aphasia assessment	Computerised software (ASR)	Portuguese
(Baker et al. 2023)	Observational/analytical	181	Aphasia assessment	Artificial intelligence	English
(Ballard et al. 2019)	Experimental	5	Aphasia assessment	Computerised software	English
(Cong et al. 2024)	Observational/analytical	782	Aphasia assessment	Artificial intelligence	English
(Day et al. 2021)	Observational/analytical	238	Aphasia assessment	Artificial intelligence	English
(Dupuis Desroches et al. 2026)	Observational/analytical	828	Aphasia assessment	Artificial intelligence	English & French
(Fergadiotis et al. 2019)	Controlled clinical trial	47	Aphasia assessment	Computerised software	English
(Fontan et al. 2024)	Observational/analytical	34	Aphasia assessment	Artificial intelligence	French
(Herath et al. 2022)	Observational/analytical	54	Aphasia assessment	Artificial intelligence	English
(Ivanova et al. 2021)	Experimental	191	Aphasia assessment	Computerised software	Russian
(Kristinsson et al. 2020)	Experimental	116	Aphasia assessment	Artificial intelligence	English
(Le et al. 2016)	Observational/analytical	31	Aphasia assessment	Artificial intelligence	English
(Le et al. 2018)	Observational/analytical	588	Aphasia assessment	Artificial intelligence	English
(Mahmoud et al. 2021)	Observational/analytical	46	Aphasia assessment	Artificial intelligence	Mandarin
(Metu et al. 2024)	Observational/analytical	34	Aphasia assessment	Artificial intelligence	English
(Newton et al. 2013)	Experimental	15	Aphasia assessment	Computerised software	English
(Qin et al. 2020)	Observational/analytical	210	Aphasia assessment	Artificial intelligence	Cantonese
(Vallila‐Rohter et al. 2018)	Experimental	50	Aphasia assessment	Computerised software	English
(Vaitheeshwari et al. 2026)	RCT	30	Aphasia assessment; Language production & comprehension	Virtual reality + Artificial intelligence	Mandarin
(Wall et al. 2018)	Experimental	96	Aphasia assessment	Virtual reality	English
(Atalık and Kayıkcı 2025)	Experimental	7	Word finding	Computerised software	Turkish
(Cancer et al. 2026)	RCT	9	Word finding	Virtual reality	Italian
(González‐Sánchez and Vereda Alonso 2025)	Pre‐post cohort	10	Word finding	Computerised software	Spanish
(Grechuta et al. 2016)	Case series	2	Word finding; Language production	Virtual reality	Spanish
(Grechuta et al. 2019)	RCT	17	Word finding	Virtual reality	Spanish
(Grechuta et al. 2020)	Experimental	10	Word finding	Virtual reality	Spanish
(Harrison et al. 2023)	Experimental	83	Word finding	Computerised software	English
(Joubert et al. 2024)	Single‐case experimental	1	Word finding	Computerised software	French
(Kurland et al. 2014)	Experimental	8	Word finding	Computerised software	English
(Kurland et al. 2018)	Experimental	21	Word finding	Computerised software	English
(Kurul et al. 2026)	RCT	92	Word finding	Virtual reality	Turkish
(Lavoie et al. 2018)	Case series	4	Word finding	Computerised software	French
(Marshall et al. 2013)	Experimental	9	Word finding	Computerised software	English
(Palmer et al. 2012)	RCT	34	Word finding	Computerised software	English
(Palmer et al. 2019)	RCT	278	Word finding	Computerised software	English
(Robinaugh et al. 2024)	Single‐case experimental	1	Word finding	Computerised software	English
(Routhier et al. 2016)	Case series	2	Word finding	Computerised software	French
(Upton et al. 2024)	RCT	27	Word finding	Computerised software	English
(Burdea et al. 2015)	Case study	1	Language comprehension	Virtual reality	English
(Cherney and Van Vuuren 2022)	Experimental	16	Language production	Virtual reality	English
(Cherney et al. 2021)	RCT	32	Language production & comprehension	Virtual reality	English
(De Luca et al. 2018)	RCT	32	Language production & comprehension	Computerised software	Italian
(De Luca et al. 2023)	Controlled clinical trial	30	Language comprehension; Reading; Writing	Virtual reality	Italian
(Des Roches et al. 2015)	Prospective single‐group design	51	Language production; Reading; Writing	Computerised software	English
(Elhakeem et al. 2021)	RCT	50	Language production & comprehension	Computerised software	Arabic
(Giachero et al. 2020)	RCT	36	Language comprehension; Word finding	Virtual reality	Italian
(Grasso et al. 2025)	Pre‐post group analysis (single‐subject multiple baseline)	20	Language production	Computerised software	English
(Imaezue 2025)	Single‐case crossover (counterbalanced)	3	Language production	Computerised software	English
(Kesav et al. 2017)	RCT	20	Language comprehension; Reading	Computerised software	Malayalam
(Krzok et al. 2017)	Experimental	16	Language production & comprehension	Computerised software	German
(Liu et al. 2021)	Controlled clinical trial	68	Language production & comprehension	Computerised software	Chinese
(Marshall et al. 2023)	Case series	2	Language production	Virtual reality	English
(Ruiz Ares et al. 2025)	Experimental	40	Language production & comprehension	Computerised software	Spanish
(Spaccavento et al. 2021)	Pilot non‐inferiority	22	Language production; Writing	Computerised software	Italian
(Stark and Warburton 2018)	Experimental	10	Language production	Computerised software	English
(Wischmann et al. 2025)	RCT	104	Language production & comprehension	Computerised software	German
(Braun and Cherney 2025)	Experimental	10	Functional communication	Computerised software	English
(Cetinkaya et al. 2024)	Experimental	5	Functional communication; Reading; Writing; Language production & comprehension	Computerised software	English
(Cherney et al. 2019)	Experimental	20	Functional communication	Virtual reality	English
(Kalinyak‐Fliszar et al. 2015)	Experimental	4	Functional communication	Virtual reality	English
(Marshall et al. 2016)	Quasi‐RCT	20	Functional communication	Virtual reality	English
(Marshall et al. 2020)	RCT	34	Functional communication	Virtual reality	English
(Cancer et al. 2026)	Single‐case pilot	2	Reading	Computerised software	Italian
(Caute et al. 2019)	Quasi‐RCT	21	Reading	Computerised software	English
(Donoso Brown et al. 2025)	Non‐concurrent multiple baseline SCED	6	Reading	Computerised software (mobile app + self‐monitoring adjuncts)	English
(Lavoie et al. 2015)	Case study	1	Writing	Computerised software	French
(Marshall et al. 2018)	Quasi‐RCT	21	Writing	Computerised software	English

### 3.2 Sample Size

Sample sizes varied considerably across the 67 included studies. The majority involved fewer than 50 participants, accounting for 73.13% of studies (*n* = 49). Studies with medium sample sizes, ranging from 50 to 200 participants, represented 17.91% (*n* = 12), while 8.96% (*n* = 6) involved over 200 participants. It is worth noting that, with the exception of Palmer et al. (2019), the larger samples are predominantly found among the assessment studies. This reflects the methodological approach common to AI‐based assessment research, where studies frequently drew on pre‐existing labelled speech corpora, most notably AphasiaBank, rather than recruiting new participants, making large sample sizes more feasible than in controlled intervention trials (**as shown in** **Table** **1**).

### 3.3 Languages Used Across Studies

Most of the studies were conducted in English, 39 out of 67 (58.21%). Italian and French were both represented in six studies each (8.96%), followed by Spanish in five studies (7.46%). Turkish, German and Mandarin were each used in two studies (2.99%), while Portuguese, Arabic, Russian, Malayalam, Chinese and Cantonese were each used in a single study (1.49%) (**as shown in** **Table** **1**). One study was bilingual, incorporating both English and French stimuli and participants (Dupuis Desroches et al. 2026). This distribution highlights a continued predominance of English‐speaking populations in the literature, though the range of languages represented has broadened in comparison to earlier reviews in this field. Furthermore, two studies conducted in languages other than English used English‐based assessments as outcome measures, namely, the Western Aphasia Battery and the Boston Naming Test, which were translated or adapted for Mandarin by Liu et al. (2021) and Arabic by Elhakeem et al. (2021).

### 3.4 Adherence to Recommended Dose for the Intervention Targets (per %)

Across the therapy‐focused studies, dose varied considerably by intervention target. Reading showed the lowest adherence to the recommended 20‐h threshold, with 33.3% of studies with reported dose meeting this benchmark (*n* = 2 of 6). Functional communication reached 50.0% (*n* = 3 of 6), writing 60.0% (*n* = 3 of 5), language production and comprehension 68.4% (*n* = 13 of 19) and word finding showed the highest adherence at 70.6% (*n* = 12 of 17). Studies where dose was not reported are excluded from these calculations. These patterns suggest that underdosing remains a challenge across all intervention targets and may partly account for the variable effectiveness observed in domains with lower dose adherence, as shown in **Table** **2**.

**TABLE 2:** Dosage for therapy‐focused studies.

Intervention target	Study ID	Dose (total hours)	Meets ≥20 h (RELEASE, 2022)
Writing			
	(Spaccavento et al. 2021)	33 h	Yes
	(De Luca et al. 2023)	18 h	No
	(Des Roches et al. 2015)	40 h	Yes
	(Lavoie et al. 2015)	NR	Uncertain
	(Marshall et al. 2018)	12 h	No
	(Cetinkaya et al. 2024)	∼36.5 h	Yes
Reading			
	(De Luca et al. 2023)	18 h	No
	(Des Roches et al. 2015)	40 h	Yes
	(Kesav et al. 2017)	12 h	No
	(Caute et al. 2019)	14 h	No
	(Cancer et al. 2026)	5 h	No
	(Cetinkaya et al. 2024)	36.5 h	Yes
	(Donoso Brown et al. 2025)	NR	Uncertain
Language production and comprehension			
	(Cherney and Van Vuuren 2022)	27 h	Yes
	(Cherney et al. 2021)	54 h	Yes
	(Burdea et al. 2015)	NR	Uncertain
	(De Luca et al. 2018)	18 h	No
	(Des Roches et al. 2015)	40 h	Yes
	(Elhakeem et al. 2021)	48 h	Yes
	(Grechuta et al. 2016)	27 h	Yes
	(Krzok et al. 2017)	32 h	Yes
	(Liu et al. 2021)	12 h	No
	(Marshall et al. 2023)	20 h	Yes
	(Spaccavento et al. 2021)	33 h	Yes
	(Stark and Warburton 2018)	9.5 h	No
	(De Luca et al. 2023)	18 h	No
	(Giachero et al. 2020)	96 h	Yes
	(Kesav et al. 2017)	12 h	No
	(Ruiz Ares et al. 2025)	≥45 h	Yes
	(Wischmann et al. 2025)	<20 h supervised (acute setting)	No
	(Grasso et al. 2025)	∼20–24 h	Yes
	(Imaezue 2025)	30 h	Yes
	(Vaitheeshwari et al. 2026)	NR	Uncertain
	(Cetinkaya et al. 2024)	36.5 h	Yes
Functional communication			
	(Kalinyak‐Fliszar et al. 2015)	2 h	No
	(Marshall et al. 2016)	25 h	Yes
	(Marshall et al. 2020)	21 h	Yes
	(Cherney et al. 2019)	1 h	No
	(Braun and Cherney 2025)	2 h	No
	(Cetinkaya et al. 2024)	36.5 h	Yes
Word finding			
	(Palmer et al. 2012)	25 h	Yes
	(Marshall et al. 2013)	15 h	No
	(Kurland et al. 2014)	48 h	Yes
	(Grechuta et al. 2016)	27 h	Yes
	(Routhier et al. 2016)	20 h	Yes
	(Kurland et al. 2018)	9 h	No
	(Grechuta et al. 2019)	23 h	Yes
	(Lavoie et al. 2018)	13.5 h	No
	(Grechuta et al. 2020)	20 h	Yes
	(Harrison et al. 2023)	28 h	Yes
	(Upton et al. 2024)	60 h	Yes
	(Palmer et al. 2019)	28 h	Yes
	(Giachero et al. 2020)	96 h	Yes
	(Atalık and Kulak Kayıkcı 2025)	NR	Uncertain
	(Robinaugh et al. 2024)	8 h	No
	(Cancer et al. 2026)	10 h	No
	(González‐Sánchez and Vereda Alonso 2025)	30 h	Yes
	(Kurul et al. 2026)	40 h	Yes
	(Joubert et al. 2024)	NR	Uncertain

*Notes*: Several studies appear more than once because they evaluated more than one intervention target. Studies with partial or incomplete dosage information are noted.

Abbreviation: NR, not reported.

## 4 Risk of Bias and Certainty of Evidence Results

Across the 67 included studies, 7.5% were rated High quality (*n* = 5), 41.8% Moderate (*n* = 28) and 50.7% Low (*n* = 34) indicating that the majority of the included studies have a moderate to high risk of bias (**see** **Table** **3**). Examining the pattern of ratings across the five NICE domains reveals both strengths and consistent limitations in the included literature. The outcomes domain was the most consistently well‐addressed, with 80.6% of studies rated as effectively reducing potential for bias in this domain (*n* = 54). By contrast, the analysis and summary domains were the most problematic, with notable bias ratings recorded in 14.9% (*n* = 10) and 10.4% (*n* = 7) of studies respectively, most commonly reflecting limited blinding, inadequate statistical reporting and insufficient description of analytical procedures. The high‐quality studies, all RCTs with adequate randomisation and analytical rigour (Elhakeem et al. 2021; Harrison et al. 2023; Kurul et al. 2026; Liu et al. 2021; Palmer et al. 2019), carry the greatest weight in the GRADE certainty assessments (**see** **Table** **4**). Six intervention targets were identified across the included studies: technology‐based assessment accuracy, word finding, language production and comprehension, functional communication, reading and writing.

**TABLE 3:** Risk of bias results following NICE guidelines.

Study	Population	Methods	Outcomes	Analysis	Summary	Quality rating
(Abad et al. 2013)	+	+	++	+	+	Low
(Atalık and Kulak Kayıkcı 2025)	+	+	+	+	+	Low
(Baker et al. 2023)	++	+	++	++	+	Moderate
(Ballard et al. 2019)	+	+	++	+	+	Low
(Braun and Cherney 2025)	+	++	+	++	+	Moderate
(Burdea et al. 2015)	+	+	+	—	—	Low
(Cancer et al. 2026)	+	+	+	+	+	Low
(Cancer et al. 2026)	+	+	++	—	+	Low
(Caute et al. 2019)	+	+	+	+	+	Low
(Cetinkaya et al. 2024)	+	+	++	+	+	Low
(Cherney and Van Vuuren 2022)	++	+	++	+	+	Moderate
(Cherney et al. 2019)	+	+	++	++	+	Moderate
(Cherney et al. 2021)	++	+	++	+	+	Moderate
(Cong et al. 2024)	++	+	++	+	+	Moderate
(Day et al. 2021)	++	+	++	++	+	Moderate
(De Luca et al. 2018)	+	+	++	+	+	Low
(De Luca et al. 2023)	+	+	++	++	+	Moderate
(Des Roches et al. 2015)	++	+	++	+	+	Moderate
(Donoso Brown et al. 2025)	—	+	+	+	+	Low
(Dupuis Desroches et al. 2026)	++	+	++	++	+	Moderate
(Elhakeem et al. 2021)	++	++	++	++	++	High
(Fergadiotis et al. 2019)	++	+	++	++	+	Moderate
(Fontan et al. 2024)	+	+	++	+	+	Low
(Giachero et al. 2020)	+	++	++	+	+	Moderate
(González‐Sánchez and Vereda Alonso 2025)	+	+	++	+	+	Low
(Grasso et al. 2025)	+	++	++	++	+	Moderate
(Grechuta et al. 2016)	+	+	++	—	—	Low
(Grechuta et al. 2019)	++	+	++	—	—	Low
(Grechuta et al. 2020)	++	+	++	—	+	Low
(Harrison et al. 2023)	++	++	++	++	++	High
(Herath et al. 2022)	++	+	+	+	+	Low
(Imaezue 2025)	+	++	++	++	+	Moderate
(Ivanova et al. 2021)	++	+	++	+	++	Moderate
(Joubert et al. 2024)	+	+	+	+	+	Low
(Kalinyak‐Fliszar et al. 2015)	+	+	++	—	—	Low
(Kesav et al. 2017)	+	+	++	+	+	Low
(Kristinsson et al. 2020)	++	+	++	+	+	Moderate
(Krzok et al. 2017)	+	+	++	++	+	Moderate
(Kurland et al. 2014)	+	+	++	+	—	Low
(Kurland et al. 2018)	+	+	++	+	+	Low
(Kurul et al. 2026)	++	++	++	++	++	High
(Lavoie et al. 2015)	+	+	+	+	+	Low
(Lavoie et al. 2018)	+	+	++	+	+	Low
(Le et al. 2016)	+	+	++	—	+	Low
(Le et al. 2018)	++	+	+	++	+	Moderate
(Liu et al. 2021)	++	++	++	++	++	High
(Mahmoud et al. 2021)	+	+	++	++	++	Moderate
(Marshall et al. 2013)	+	+	++	+	—	Low
(Marshall et al. 2016)	++	+	++	+	+	Moderate
(Marshall et al. 2018)	+	+	++	++	+	Moderate
(Marshall et al. 2020)	+	++	++	+	+	Moderate
(Marshall et al. 2023)	+	+	++	—	—	Low
(Metu et al. 2024)	+	+	++	+	+	Low
(Newton et al. 2013)	+	++	+	+	+	Low
(Palmer et al. 2012)	+	++	++	++	+	Moderate
(Palmer et al. 2019)	++	++	++	++	++	High
(Qin et al. 2020)	++	+	++	+	+	Moderate
(Robinaugh et al. 2024)	+	+	+	++	+	Low
(Routhier et al. 2016)	+	+	++	—	+	Low
(Ruiz Ares et al. 2025)	+	+	+	+	+	Low
(Spaccavento et al. 2021)	++	+	++	—	+	Low
(Stark and Warburton 2018)	+	+	++	++	+	Moderate
(Upton et al. 2024)	+	+	++	++	++	Moderate
(Vaitheeshwari et al. 2026)	+	+	++	++	+	Moderate
(Vallila‐Rohter et al. 2018)	+	+	++	+	+	Low
(Wall et al. 2018)	+	+	++	+	+	Low
(Wischmann et al. 2025)	+	+	++	++	+	Moderate

Key: ++ = Study design effectively reduces potential for bias | + = Unclear or may not have fully addressed all potential sources of bias | − = Notable sources of bias present.

**TABLE 4:** GRADE result for intervention targets/assessment accuracy.

Intervention target/Assessment accuracy	Number of studies	Certainty of evidence	Comments
Technology‐based assessment	21	Moderate	Many studies were well‐designed with large sample sizes, though a number drew on pre‐existing labelled speech corpora rather than prospectively recruited samples, and lack of blinding was common. Reported classification accuracies were sometimes very high, including figures approaching 99 % in some models, but these require cautious interpretation given small or specific datasets, limited external validation and limited evidence of clinical generalisability. Outcomes varied considerably by technology type, with AI and ASR approaches differing in clinical applicability. High classification accuracy does not yet translate to demonstrated patient‐level benefit in real life clinical context.
Word finding improvement	21	High	The strongest and most consistent body of evidence in this review. Most studies reported meaningful improvements in naming abilities across both CSLT and VR approaches, with only Marshall et al. (2013) failing to show significant change. The majority delivered therapy at or above the recommended 20‐h threshold, and higher‐dose studies were consistently associated with stronger and more generalisable gains. Generalisation to untrained items remained more modest and variable. High certainty is maintained, driven by consistent evidence from multiple RCTs, though risk of bias ratings varied across studies, with some RCTs demonstrating lower risk of bias than others.
Language production and comprehension	21	Moderate	Most interventions produced significant within‐group gains on expressive language measures, but between‐group superiority over face‐to‐face SLT was less consistent. Approximately half of the studies met the recommended 20‐h threshold, with higher‐dose studies generally reporting clearer gains. Two studies targeted the acute post‐stroke phase, where effects cannot always be separated from spontaneous recovery. Certainty remains Moderate given variability in designs, populations, outcome measures and dose adherence.
Functional communication	6	Moderate	Findings were mixed, reflecting predominantly moderate‐quality studies with some risk of bias from limited randomisation or blinding. All studies had small sample sizes, most under 40 participants, reducing confidence in the results. Studies using virtual environments, virtual clinician dialogue and computer‐based script training yielded measurable improvements and high engagement. Approximately half met the recommended dose threshold, with higher‐intensity studies reporting more consistent gains.
Reading skills improvement	7	Moderate	Studies were small and heterogeneous in design, resulting in imprecise effect estimates. Some demonstrated significant improvements on standardised comprehension measures; others showed no advantage over standard care. A minority of studies met the recommended 20‐h dose threshold.
Writing skills improvement	6	Low	Studies had small samples and limited blinding, with considerable variability in the extent of gains. Task‐specific improvements were more commonly observed than generalised gains across writing sub‐skills. Around 50% of studies met the recommended dose threshold. Low certainty reflects small samples, limited blinding, inconsistent outcome breadth and the absence of large, controlled trials.

*Note*: Some studies evaluated more than one intervention target.

### 4.1 Types of Technological Solutions

A range of technologies have been used in aphasia rehabilitation studies, each offering distinct features and applications. Although terminology may vary across studies, the key approaches can be broadly categorised into three main types: computerised SLT (38 studies, 56.72%), VR (17 studies, 25.37%) and AI (13 studies, 19.40%). One study employed a combination of VR and AI (Vaitheeshwari et al. 2026) and is therefore reflected in the counts for both categories. The following sub‐sections introduce these technologies, outlining their key features and differences in application within the included studies.

### 4.2 Computerised Speech and Language Therapy

CSLT provides flexible and intensive therapy, allowing patients to practise language tasks independently while SLTs oversee the progress remotely. CSLT platforms are designed to provide high‐intensity therapy, offering patients the ability to repeat exercises as often as necessary. For instance, in the Big CACTUS trial, CSLT enabled long‐term post‐stroke aphasia participants to engage in customised daily language tasks, showing that self‐directed practice can complement face‐to‐face therapy (Palmer et al. 2019). A key feature of the systems in the studies was the ability to deliver tailored exercises targeting individual language impairments (e.g., Elhakeem et al. 2021). Across the 38 CSLT studies in this review, a variety of platforms have been used to deliver therapy, including smartphones and tablets, which enabled PWA to engage in self‐administered therapy across different platforms (**see** **Table** **1**).

Beyond therapy delivery, CSLT has also been applied to language assessment. Some CSLT systems include standardised assessments, such as the adaptive Philadelphia Naming Test‐CAT, which adjusts item difficulty in real‐time based on the user's responses. Some authors argued that this adaptability of computer‐based assessments may reduce cognitive load and improve diagnostic precision, which makes the experience less demanding for PWA (Fergadiotis et al. 2019; Newton et al. 2013). In short, CSLT represents the most widely used technology in this review, functioning both as a treatment delivery platform and as an assessment tool, with its key strength lying in its capacity to support high‐intensity, self‐directed practice at a scale that may not be feasible in routine clinical settings.

### 4.3 Virtual Reality

Across the VR studies in this review, the technology was predominantly applied to functional communication and word finding, with immersive and semi‐immersive formats offering distinct but complementary approaches to language practice for PWA. VR offers an opportunity for an immersive, interactive experience for PWA. Unlike face‐to‐face therapy approaches, VR allows patients to engage in simulated environments that imitate real‐world communication scenarios​ (Kalinyak‐Fliszar et al. 2015). For example, EVA Park is a virtual world designed specifically for PWA, offering a range of real‐world locations such as cafes, health centres and restaurants (Marshall et al. 2016). This environment provides a space where patients can practise communication skills with others in a virtual context. The interaction occurs through avatars, allowing users to communicate via speech or typing, aiming to reduce feelings of embarrassment and increasing motivation for language practice (Marshall et al. 2020). Kalinyak‐Fliszar et al. (2015), introduced a virtual clinician (VC) to guide participants through scripted dialogues aimed at supporting functional communication. This approach can allow PWA to practise everyday communication tasks in a structured and consistent way, without requiring continuous involvement from an SLT. In addition, features such as motion tracking and real‐time feedback, may contribute to making therapy more interactive and potentially adaptable to individual needs (De Luca et al. 2023). On the other hand, fully immersive headset‐based VR has also been explored; Kurul et al. (2026) conducted the first large RCT using immersive VR for naming therapy, demonstrating significantly greater gains than face‐to‐face therapy alone. It is worth noting that immersive VR, like CSLT, may also function partly as a dose‐enabling tool, allowing PWA to engage in greater amounts of self‐directed naming practice than face‐to‐face therapy permits.

### 4.4 Artificial Intelligence

AI refers to the capability of machines and computer systems to perform tasks typically associated with human intelligence, such as learning, problem‐solving, decision‐making and perception (Russell and Norvig 2020). Its introduction into aphasia assessment and treatment began with the development of Automatic Speech Recognition (ASR) technology, which is designed to convert speech into text. Early work in this area demonstrated that ASR could be used to support word‐naming therapy tasks, enabling systems to detect correct or incorrect word retrieval attempts (Abad et al. 2013). Although these approaches showed promise for delivering automated assessment, they were hampered by the atypical and variable nature of aphasic speech, which poses a persistent challenge for automated recognition systems.

Building on these early developments, ASR systems were extended from isolated word recognition to the processing of longer utterances and narratives. This shift was particularly important for clinical contexts, where the analysis of spontaneous speech provides different but potentially valuable diagnostic information. For example, Qin et al. (2020) developed a deep neural network‐based ASR system trained on speech from Cantonese speakers with and without aphasia, demonstrating that automated transcription could be combined with story‐level embeddings and Siamese networks to assess discourse‐level impairments.

Further advances in AI have been driven by the rapid development of Large Language Models (LLMs), which extend the analysis of spoken language beyond transcription by enabling detailed examination of word and sentence‐level deficits. For instance, Cong et al. (2024) demonstrated that LLMs can identify aphasia‐related deficits and improve diagnostic accuracy when integrated with existing language indices. At the same time, broader applications of AI in aphasia have focused on automating core assessment tasks such as subtype identification, severity classification and prediction of recovery outcomes. For example, Herath et al. (2022) developed a speech‐based classification system using deep neural networks to distinguish between levels of aphasia severity. Similarly, Mahmoud et al. (2021) compared classical machine‐learning frameworks with convolutional neural network approaches, showing that deep learning methods more effectively captured the complexity of aphasic language. Extending this line of research, Kristinsson et al. (2020) applied support vector regression to multimodal neuroimaging data to predict aphasia severity and language performance, highlighting the potential of machine‐learning models to support outcome prediction.

However, the application of AI in clinical contexts raises important considerations. Many models are trained on data drawn from specific linguistic and demographic groups, which may limit their generalisability across languages, aphasia types and clinical settings, as evidenced by the substantially higher error rates observed for PWA compared to neurologically healthy speakers and across different languages (Dupuis Desroches et al. 2026). Furthermore, concerns around model explainability and algorithmic transparency (Holzinger et al. 2019), as well as the ethical use of patient speech data including informed consent and data protection (Char et al. 2018), warrant careful consideration before integration to clinical practice.

### 4.5 Accuracy of Technology in the Assessment of PWA

A total of 21 studies examined the use of technology for the assessment and classification of aphasia, spanning a range of approaches from automatic speech recognition to natural language processing and computerised adaptations of established assessment batteries (as presented in Table 1). The majority focused on evaluating whether automated systems could reliably distinguish PWA from neurologically typical speakers, classify aphasia subtypes or severity, or digitalise some existing assessment procedures. Across these studies, three broad approaches were represented: automatic speech recognition (ASR), natural language processing (NLP) and computerised adaptations of established assessment tools.

ASR‐based systems showed considerable variation in performance depending on the task and speech material. Error rates for continuous recognition of aphasic speech remained substantial across studies, reflecting the fundamental challenge that atypical, and phonologically variable speech poses for systems trained predominantly on neurotypical speakers. For instance, Le et al. (2018) reported a word error rate of approximately 37% for continuous speech, and Qin et al. (2020) reported a comparable error rate for syllable recognition on narrative tasks, both indicating the significant challenge of recognising speech from PWA. Recently, Dupuis Desroches et al. (2026), the largest ASR benchmarking study in this review, demonstrated that current models perform up to 20 percentage points worse for PWA than for neurologically healthy speakers, with clinically important features such as filled pauses almost entirely missed by existing systems. These findings temper the more optimistic word‐level agreement figures reported in earlier studies such as Ballard et al. (2019), who found approximately 80% agreement between ASR judgements and clinician ratings at the word level, and collectively highlight that laboratory performance does not straightforwardly translate to clinical utility.

NLP approaches demonstrated a wider range of applications, from severity classification and subtype identification to discourse‐level analysis and recovery prediction. Reported accuracies varied considerably across tasks and models, ranging from approximately 67% for multimodal outcome prediction (Kristinsson et al. 2020) to over 90% for binary aphasia detection (Cong et al. 2024) and severity classification (Herath et al. 2022). However, these figures require careful contextualisation. The majority of reported accuracies were obtained by testing models against pre‐existing labelled datasets, most notably AphasiaBank, which is predominantly English‐speaking and drawn from specific demographic groups. Models achieving very high classification accuracies, including figures approaching 99% in some instances (Herath et al. 2022), were typically tested on small, carefully curated datasets under controlled conditions, and none were evaluated in prospective clinical settings. The validity of these systems was assessed against SLT‐labelled data, which represents an important methodological strength; however, the gap between classification performance on labelled corpora and real‐world clinical deployment remains substantial and largely unexamined.

Computerised adaptations of established assessment batteries offered more clinically proximal evidence. Studies examining tablet‐based and computer‐delivered versions of tools such as the Philadelphia Naming Test (Fergadiotis et al. 2019), the Western Aphasia Battery (Vallila‐Rohter et al. 2018) and the Russian Aphasia Test (Ivanova et al. 2021) consistently demonstrated strong agreement with their pen‐and‐paper counterparts, alongside practical advantages including reduced administration time, improved documentation completeness, and higher completion rates among stroke survivors (Wall et al. 2018). These findings suggest that computerised assessment tools can meaningfully support clinical workflows, particularly in acute and under‐resourced settings. However, patient preferences were not uniformly positive, as Newton et al. (2013) found that participants generally scored lower on computerised tasks and expressed a preference for the presence of an SLT, a finding that serves as an important reminder that feasibility and acceptability are not equivalent.

Across all 21 assessment studies, none demonstrated that automated systems could replicate the full breadth of clinical aphasia assessment, with gesture, pragmatics and connected discourse comprehension remaining beyond the reach of current technology.

### 4.6 The Effectiveness of Aphasia Treatment When Delivered Using Technology

This section is divided into six subsections according to the language domain treated (intervention target): word finding, language production, language comprehension, functional communication, reading and writing skills.

## 5 Word Finding

Word finding was the most extensively investigated intervention target in this review, with 19 studies across CSLT and VR approaches, and the highest certainty of evidence of any domain. The evidence consistently demonstrates that technology‐based interventions produce meaningful improvements in naming for trained targets when delivered at adequate intensity. Across both CSLT and VR modalities, studies meeting or exceeding the recommended 20‐h threshold reported significant gains in trained‐word retrieval (e.g., Palmer et al. 2019; Kurland et al. 2014; Grechuta et al. 2019; Kurul et al. 2026). By contrast, studies delivering below this threshold were more likely to report limited or absent improvement, including modest severity‐dependent gains at 9 h (Kurland et al. 2018), and no significant change at 15 h (Marshall et al. 2013). This dose‐response pattern held across both technology types, which may suggest that technology functions primarily as a tool to make high‐intensity naming practice feasible and sustainable for PWA rather than a shortcut to effective therapy.

Both CSLT and VR produced comparable patterns of gain for trained items, though their mechanisms differ. CSLT studies predominantly used personalised word lists and cueing hierarchies to target individual lexical deficits (e.g., Lavoie et al. 2018; Harrison et al. 2023; Upton et al. 2024; González‐Sánchez and Vereda Alonso 2025), while VR studies embedded naming within communicative exchanges and multisensory environments (Grechuta et al. 2016, 2019; Grechuta et al. 2020; Kurul et al. 2026). Despite these differences in approach, the direction of findings was consistent across both modalities at sufficient dose, with high‐quality RCTs in each demonstrating superiority over face‐to‐face therapy (Palmer et al. 2019; Kurul et al. 2026).

Generalisation to untrained items was the exception rather than the rule across the evidence base. The majority of studies reported item‐specific gains without significant transfer beyond trained targets (e.g., Upton et al. 2024; Grechuta et al. 2020), which is consistent with the broader word retrieval literature. Only two studies demonstrated statistically significant generalisation to untrained words, both delivered at or above the recommended dose threshold (Atalık and Kulak Kayıkcı 2025; González‐Sánchez and Vereda Alonso 2025). This may suggest that higher‐intensity protocols may be necessary but not sufficient for broader lexical gains.

The strongest evidence came from studies that met the dose threshold and demonstrated clear between‐group superiority (Palmer et al. 2019; Kurul et al. 2026). Moderate and Low‐quality studies according to NICE ROB assessment also predominantly reported positive outcomes for trained items, but effect sizes were more variable, and generalisation was rarely demonstrated.

## 6 Language Production and Comprehension

Language production and comprehension was the second most extensively investigated domain in this review, with 21 studies spanning CSLT and VR approaches, and a moderate certainty of evidence (GRADE; see Table 4). Across the included studies, most technology‐based interventions produced significant within‐group gains on expressive language measures, though between‐group superiority over face‐to‐face SLT was less consistent, and findings varied considerably by dose, study quality and population.

For language production, the clearest gains were observed in studies delivering therapy at or above the recommended 20‐h threshold. Studies comparing technology‐based and therapist‐delivered approaches consistently found comparable or superior gains in the technology group, without statistically significant between‐group differences in most cases (Spaccavento et al. 2021; De Luca et al. 2018). Higher‐intensity studies showed clearer between‐group advantages: combined computer‐based and face‐to‐face SLT produced significantly greater improvements in spontaneous speech than SLT alone (WAB spontaneous speech: 13.50 to 17.50 vs. 13.18 to 14.60; *p* = 0.026; Liu et al. 2021), and software‐supported therapy yielded greater gains in phrase length than face‐to‐face SLT (2.80 vs. 2.12 words per phrase; *p* = 0.036; Elhakeem et al. 2021). Technology‐delivered script therapy produced substantial within‐group gains in spoken script production across studies (Cherney and Van Vuuren 2022; Marshall et al. 2023), and self‐delivered iPad therapy significantly increased connected‐speech content units in all participants (*p* < 0.01; Stark and Warburton 2018). Improvements were also consistently reported on expressive‐language subscales of standardised assessments including the Western Aphasia Battery and Aachen Aphasia Test across multiple studies (Grechuta et al. 2016; Cherney et al. 2021; Krzok et al. 2017). Script training in progressive aphasia produced significant improvements in trained script accuracy, speech rate and fluency disruptions, with the first group‐level evidence of generalisation to untrained topics (Grasso et al. 2025), though gains in this context represent stabilisation rather than recovery given the progressive nature of the condition. The only study targeting spontaneous unscripted speech delivered entirely at home without clinician contact found large post‐treatment effects on microlinguistic outcomes and preliminary evidence of generalisation to untreated prompts, further supporting the dose‐enabling role of technology in independent self‐management (Imaezue 2025).

Regarding language comprehension, the clearest gains were observed in VR‐based studies. De Luca et al. (2023) reported greater gains in auditory comprehension for the VR group compared with face‐to‐face SLT (mean increase approximately +7.6 vs. +2.3 points), maintained at 6‐month follow‐up (*p* < 0.001). Similarly, Giachero et al. (2020) found that language comprehension improved only in the VR group despite equivalent SLT dose across groups (AAT auditory comprehension: 38.1 to 46.4; *p* = 0.004), while Vaitheeshwari et al. (2026) demonstrated that a combined VR and AI system outperformed SLT‐only on six of nine language subtests, with the control group showing no significant gains on any subtest. By contrast, adding CSLT to face‐to‐face SLT did not produce greater comprehension gains than SLT alone in a feasibility RCT (Kesav et al. 2017), which may suggest that VR may offer advantages for comprehension through its immersive communicative contexts.

Two studies targeted the acute post‐stroke phase, where interpreting technology effects requires particular caution. Wischmann et al. (2025) conducted the first acute‐phase RCT in this domain, finding no significant difference on the primary outcome, though an adjusted analysis favoured tablet‐assisted SLT (*p* = 0.044) and subgroup analyses revealed large effect sizes in mild and moderate aphasia.

Notably, technology significantly increased self‐directed practice time (10 h vs. 4 h; *p* = 0.001), supporting its dose‐enabling role even when overall supervised dose fell below the RELEASE threshold. Ruiz Ares et al. (2025) similarly reported significant pre‐post improvements following a digital SLT tool, though the absence of a control group means these gains cannot be attributed to the technology alone, as spontaneous neurological recovery is a likely confound.

Technology‐based interventions for language production and comprehension produced meaningful gains across the included studies, particularly at sufficient dose, though between‐group superiority over face‐to‐face SLT was not consistently demonstrated. VR showed promise for comprehension outcomes, while CSLT demonstrated consistent benefits for production across diverse languages. The moderate certainty rating reflects variability in study designs, inconsistent between‐group effects, and the challenges of separating technology effects from spontaneous recovery in acute‐phase studies.

## 7 Functional Communication

Six studies examined functional communication using VR and CSLT approaches, with a Moderate certainty of evidence (GRADE; see Table 4). Across the included studies, technology‐based interventions produced measurable improvements on functional communication measures, though findings varied by approach, dose and study design.

Script‐based CSLT approaches showed consistent gains across studies delivering varying doses. Computer‐delivered script training produced immediate and maintained improvements in speech accuracy and rate, with modest generalisation to conversational accuracy on trained topics (Cherney et al. 2019). A subsequent study using the same approach demonstrated that even a precisely controlled minimal dose produced significant gains in script accuracy and rate, with meaningful generalisation to a live communication partner (Braun and Cherney 2025). Practising everyday dialogues with a virtual clinician produced outcomes comparable to face‐to‐face SLT sessions, with improvements in both informativeness and communicative efficiency (Kalinyak‐Fliszar et al. 2015).

VR‐based approaches delivered at higher intensities showed clearer and more sustained gains on standardised functional communication measures. A quasi‐RCT using EVA Park reported significant improvements in Communication Activities of Daily Living scores, maintained at follow‐up, while a waitlist group showed no change during the waiting period before receiving the intervention (Marshall et al. 2016). A subsequent RCT using the same VR environment demonstrated high participant engagement alongside significant within‐group gains in overall language ability (Marshall et al. 2020). A multi‐domain CSLT programme delivered above the recommended dose threshold produced significant improvements in functional communication confidence across all participants (Cetinkaya et al. 2024).

Technology‐based interventions for functional communication produced measurable gains across script training, virtual clinician dialogue and immersive VR environments. Higher‐intensity studies reported more consistent effects, while lower‐dose studies tended to show immediate but less durable gains. It is worth noting that the variability in therapist involvement and communicative context across studies makes it difficult to attribute improvements solely to the technology itself. All studies had small sample sizes, and the evidence base remains predominantly of Moderate quality, limiting the confidence with which findings can be generalised to broader clinical populations.

## 8 Reading

Seven studies examined reading using CSLT and VR approaches, with a Moderate certainty of evidence (see Table 4). Findings were mixed across the included studies, with technology showing clearest advantages when delivered with aphasia‐friendly adaptations and at sufficient doses.

Studies with active comparators provided the most informative evidence. A controlled study comparing VR‐based training with face‐to‐face SLT found improvements on a standardised reading subtest maintained at 6‐month follow‐up in the VR group, while the face‐to‐face group remained stable across all timepoints (De Luca et al. 2023). Additionally, a prospective study evaluating iPad‐based therapy with additional home practice found that greater app use was associated with larger gains in task accuracy and more positive changes on standardised assessments compared to a clinic‐only group (Des Roches et al. 2015). A quasi‐RCT evaluating tablet‐based reading therapy within an aphasia‐friendly interface demonstrated improvements in reading from baseline to post‐treatment and follow‐up, whereas a standard paper‐based comparator without aphasia‐friendly supports showed no meaningful changes (Caute et al. 2019). However, as the comparator lacked aphasia‐friendly adaptations, the observed advantage cannot be attributed to the technology alone, and the contribution of accessibility features cannot be disentangled from the technology effect.

Preliminary evidence from smaller studies added to the emerging picture. A pilot study applying rhythmic reading training in post‐stroke aphasia demonstrated encouraging effect sizes for reading speed maintained at follow‐up, despite a sub‐threshold dose, representing early feasibility evidence for this novel approach in an aphasia population (Cancer et al. 2026). A multi‐domain programme delivered above the recommended dose threshold produced improvements in reading outcomes across all participants, though effect sizes were modest and no control condition was included (Cetinkaya et al. 2024).

One study examined adherence to a home reading programme rather than reading improvement directly, finding that an aphasia‐friendly self‐monitoring logbook increased practice adherence in some participants, with no consistent improvement in reading. These findings may highlight the broader challenge of achieving sufficient home practice dose and suggests that adherence supports may be most effective when introduced early in a home‐based programme (Donoso Brown et al. 2025).

The evidence for technology‐based reading interventions remains mixed, with clearer benefits observed when interventions incorporated aphasia‐friendly adaptations and were delivered at adequate dose. Only a minority of studies met the recommended threshold, and this may partly explain the variable gains observed across the domain. The moderate certainty rating reflects small samples, heterogeneous designs and the difficulty of isolating technology effects from the influence of accessibility adaptations and therapist involvement.

## 9 Writing

Six studies examined writing using CSLT and VR approaches, with a low certainty of evidence reflecting small samples, limited blinding and inconsistent outcome breadth (**see** **Table** **4**).

Studies with active comparators suggested that technology‐based writing therapy may produce gains comparable to face‐to‐face SLT, though the evidence does not support clear superiority. A pilot non‐inferiority study found statistically significant pre‐to‐post improvements in writing across both computer‐mediated and therapist‐mediated groups, with no significant between‐group difference (Spaccavento et al. 2021). Similarly, a controlled study found closely comparable gains in writing accuracy across technology‐based and face‐to‐face groups, with no significant between‐group difference reported (De Luca et al. 2023). A prospective single‐group study reported gains for both trained and untrained items following iPad‐based therapy, suggesting some degree of generalisation beyond directly practised material, though the absence of a control group limits interpretation (Des Roches et al. 2015).

Studies targeting specific writing sub‐skills demonstrated task‐specific rather than generalised improvements. A tablet‐based intervention targeting verb writing produced substantial gains with cueing support but more modest gains without, indicating that improvements remained partially dependent on external support (Lavoie et al. 2015). No significant improvement in spelling or grammar accuracy was observed in a tablet‐based intervention targeting sentence construction, despite measurable gains on other outcome dimensions (Marshall et al. 2018). A multi‐domain programme similarly produced non‐significant spelling gains, consistent with the pattern of variable and modest writing outcomes observed across the domain (Cetinkaya et al. 2024).

Technology‐based writing therapy showed gains broadly comparable to face‐to‐face SLT in studies with active comparators, though task‐specific improvements were more consistently observed than generalised gains across writing sub‐skills. The variability in dose adherence across studies, with only around 60% of studies with reported dose meeting the recommended threshold, may partly account for the limited breadth of improvement. The Low certainty rating reflects the persistent absence of large, controlled trials, ongoing risk of bias and the need for future research with larger samples, higher doses and more comprehensive outcome measurement.

## 10 Discussion

The aim of this review was to examine the range of technologies used in aphasia assessment and therapy and to summarise their effectiveness across different intervention targets. The review found that CSLT remains the most widely used technological approach. Its prominence reflects both the maturity of the software and its capacity to deliver structured, intensive therapy at scale that is not feasible in routine clinical settings (e.g., Palmer et al. 2019). However, while most of the CSLT systems have been used to deliver repetitive practice at the recommended therapy intensities reported in this review (**see** **Table** **2**), they are primarily task‐based and less able to capture the complex nature of aphasia. VR, by contrast, was less frequently represented in the literature but offered distinct advantages. VR simulates real‐life environments and provides opportunities to practise communication in socially meaningful contexts. The use of avatars and immersive settings, as shown in EVA Park (Marshall et al. 2016), highlights how VR can address aspects of motivation, and engagement that CSLT alone may not provide. However, studies showed that VR interventions tended to be small‐scale and exploratory, and evidence for consistent gains in functional communication skills remains limited, though the recent large‐scale immersive VR RCT by Kurul et al. (2026) represents an important step toward larger‐scale evidence in this domain.

Five studies within this review examined computerised and VR approaches for aphasia assessment, showing that these systems can enhance aspects of face‐to‐face diagnostic procedures, including documentation completeness and assessment efficiency while maintaining precision. A larger subset of assessment studies applied AI‐based approaches, including automatic speech recognition, and natural language processing to classify aphasia type, severity and discourse‐level impairments, with some models achieving high classification accuracies. At the same time, the included studies point to notable constraints across both computerised and AI‐based approaches. These include performance discrepancies on computerised tasks, mixed patient preferences, reliance on carefully structured test environments, and in the case of AI, a dependence on curated datasets that limits generalisability across languages and clinical populations. Beyond accuracy, the application of AI in clinical assessment raises important considerations around model transparency, the potential for demographic bias in training data, and the ethical use of patient speech recordings, including informed consent and data protection. This positions both computerised and AI‐based assessment as developing adjuncts rather than replacements for clinician‐led evaluation, with future work needing to address ecological validity, acceptability and responsible implementation in clinical practice.

When effectiveness of technology supported SLT is considered across different intervention targets, the most robust evidence relates to word‐finding. The combined effectiveness and GRADE results may allow us to address the following questions: “does it work?” and “if so, under what conditions?”. Across the word‐finding studies, most technology‐based interventions produced clear gains in retrieval of treated words, with several RCTs and well‐designed trials showing superiority or added value over usual care, and only one study (Marshall et al. 2013) failing to show significant change. When set against the dose data, a pattern emerges: the majority of word‐finding interventions (≈70.6%) delivered at least 20 h of therapy, and the most robust effects tend to come from these higher‐intensity protocols (e.g., Upton et al. 2024), whereas studies below this threshold are more likely to show limited or task‐specific change. This aligns with broader aphasia‐dose evidence and suggests that technology is not a “shortcut” to effective naming therapy, but rather a tool that makes high‐intensity practice feasible and sustainable for PWA. On this basis and given that the certainty of evidence results (GRADE) for word‐finding is high (driven by consistent evidence from multiple RCTs), we can be reasonably confident that tech‐based interventions for word‐finding are effective when delivered at recommended intensities. However, generalisation to untrained items remained more modest and variable, so confidence is lower when extrapolating from item‐specific gains to wider communicative changes for PWA.

For language production, the evidence base is more heterogeneous than word‐finding. Across the included studies, most technology‐based interventions produced significant within‐group gains on expressive‐language measures, including improvements in spontaneous speech, and expressive subtests of standardised assessments compared to face‐to‐face therapy. However, between‐group superiority over face‐to‐face SLT was less consistent. When the effectiveness data are considered alongside dosage patterns, a similar trend to naming emerges; approximately 68.4% of language production studies met the recommended minimum of 20‐h, and these higher‐dose studies were generally those reporting clearer or more generalisable gains (e.g., Cherney et al. 2021; Giachero et al. 2020). Studies delivering lower intensities, or those relying on brief add‐on components, tended to show more modest effects or improvements limited to the practised tasks. This dose–response pattern suggests that technology can support high‐frequency practice sufficient to enhance production outcomes. The certainty of evidence for language production rated as moderate in the GRADE results, with small sample sizes and inconsistent between‐group effects limiting confidence in the generalisability of findings.

Regarding language comprehension, evidence of effectiveness is generally positive. For instance, VR‐based programmes show the clearest gains on standardised auditory‐comprehension measures, particularly when delivered at higher intensities, whereas computer add‐on approaches often produce improvements without outperforming face‐to‐face SLT. When considered with the dose data, similar to language production, around 68.4% of studies met the recommended ≥20‐h threshold, and these higher‐dose interventions were the ones most likely to show meaningful effects. The moderate certainty of evidence reflects small sample sizes and heterogeneous study designs, and the available trials are few to draw firm conclusions about whether any specific technology type offers advantages over others for language comprehension.

For functional communication, six studies demonstrated measurable improvements across script training, virtual‐clinician dialogue and VR‐based conversational environments, with gains observed on standardised functional measures including the CADL‐2 and WAB‐AQ, and high levels of participant engagement reported. Approximately half of the studies met the recommended dose threshold, and these higher‐intensity interventions were generally those reporting more consistent gains. However, these findings should be interpreted cautiously, as the evidence rests on a small number of studies with considerable variability in design, dose and the degree of therapist involvement, and the moderate certainty of evidence means that future trials could meaningfully change the current estimate.

For the effectiveness of writing, only three of the five included studies with reported dose met the recommended 20‐h threshold, which may partly account for the variability in outcomes observed across the domain. Where sufficient dose was delivered, improvements were generally limited to specific writing sub‐skills, such as verb writing, rather than generalising across broader writing abilities including spelling and grammar. Studies comparing technology‐based and therapist‐delivered writing therapy reported broadly comparable gains. The low certainty of evidence reflects the persistent influence of small samples, limited blinding and inconsistent outcome breadth across studies, and underscores the need for larger, better‐controlled trials before firm conclusions can be drawn.

Regarding reading, VR‐delivered programmes and aphasia‐friendly tablet‐based interfaces showed clearer improvements on standardised comprehension measures, whereas computer add‐on approaches did not consistently outperform face‐to‐face SLT. Dose adherence was notably low across this domain, with only two studies meeting the recommended 20‐h threshold, representing 33% of studies with reported dose. This pattern may partly explain the variable gains observed and suggests that the potential of technology‐supported reading interventions has not yet been fully tested at adequate intensity. The moderate certainty of evidence indicates that further research, particularly trials delivering higher doses with active comparators, is needed to establish the conditions under which technology‐based reading interventions can produce meaningful and generalisable gains.

A consistent methodological challenge across the included intervention domains concerns the attribution of observed effects. In most studies, technology did not function as an isolated treatment ingredient but rather as a vehicle for delivering greater amounts of practice than would be feasible through conventional face‐to‐face SLT alone. Where technology groups outperformed controls, it is therefore difficult to determine whether gains reflected the specific properties of the technology or simply the additional practice time it enabled. This distinction is particularly relevant in studies where technology substantially increased self‐directed practice time relative to control conditions (e.g., Wischmann et al. 2025), and in cases where the comparator condition lacked features present in the technology arm beyond the technology itself, such as aphasia‐friendly adaptations in reading tasks (Caute et al. 2019). Disentangling technology effects from dose effects and task adaptation effects remains an important methodological priority for future research in this field.

Although technology is increasingly presented as a means of extending access to aphasia rehabilitation, its potential to widen existing inequalities warrants consideration. Access to technology‐based interventions depends on factors including device ownership, internet connectivity, digital literacy and the availability of ongoing technical support, all of which vary considerably across age groups, socioeconomic backgrounds and geographic settings. Speech‐language pathologists have identified a multifactorial range of barriers to PWA use of technology, including age, prior technology experience, physical and cognitive factors, aphasia severity and cost of devices, alongside organisational barriers such as limited funding and restricted access to Wi‐Fi and IT infrastructure (Nichol et al. 2023). The majority of studies included in this review were conducted in hospital or university‐based settings in high‐income countries, with few reporting data on participants' prior technology experience or access to devices outside the study context, which limits the generalisability of findings to community and lower‐resource settings. Furthermore, the predominance of English‐language studies identified in this review reflects a broader inequity in the aphasia rehabilitation literature, with speakers of other languages underrepresented both in study populations and in validated technology tools (Kidd and Garcia 2022). Clinicians considering technology‐based interventions should therefore assess technology readiness and access alongside language impairment, and future research should attend explicitly to questions of equity, including the development of aphasia‐friendly, culturally appropriate interfaces and the feasibility of technology‐based interventions across diverse clinical and community settings.

The findings of this review extend and contextualise those of three prior reviews in this field. For instance, Zheng et al. (2016) reviewed seven studies of computer therapy for aphasia, concluding that CSLT was effective relative to no therapy and produced comparable outcomes to clinician‐delivered therapy, though the evidence base was small and restricted to chronic, moderate non‐fluent aphasia. The current review confirms and substantially extends this conclusion. Specifically, across 38 CSLT studies spanning a broader population and intervention range, CSLT continues to show the most consistent evidence of effectiveness, particularly for word‐finding at adequate dose. Another review conducted by Repetto et al. (2020) focused specifically on VR and mobile app interventions, identifying preliminary evidence for both approaches across 13 studies. The current review, which includes 17 VR studies, confirms these encouraging early signals while situating them within a dose framework; the conclusion that VR evidence remains exploratory and small‐scale is consistent across both reviews, though the recent large‐scale immersive VR RCT by Kurul et al. (2026) represents a meaningful step forward. Lastly, Adikari et al. (2023) conducted a review of AI applications in aphasia across 77 studies and concluded that AI was used predominantly for assessment and that therapeutic applications remained limited and not yet clinically implemented. The current review reaches a similarly cautious conclusion regarding AI for assessment and additionally situates these findings within a broader evaluation of certainty of evidence which may offer more granular picture of the conditions under which technology‐based interventions are most likely to be effective in aphasia rehabilitation.

## 11 Limitations of the Current Review

This review has several limitations that should be acknowledged. First, searches were limited to four databases (PubMed, PsycINFO, Web of Science and Scopus) without grey literature or citation tracking, which may have missed relevant studies and cannot fully exclude publication bias. Second, initial screening of all databases was conducted by the first author.  Although the third author independently screened 25% of records with high inter‐rater agreement (99.7%), this predominantly single‐reviewer approach may introduce screening bias. Third, data extraction was conducted by the first author. To minimise bias, the authorship team pre‐specified data items and handling principles for inconsistent reporting in advance. Fourth, the NICE checklist was applied uniformly across heterogeneous study designs to enable consistent comparison; however, design‐specific quality tools might have identified certain sources of bias more precisely. Fifth, heterogeneity in study designs, populations, interventions and outcome measures necessitated narrative rather than quantitative synthesis. Some quality appraisal judgements may remain subject to reviewer interpretation despite standardised tools. Finally, while teletherapy was clearly excluded, several included studies incorporated remote or hybrid therapist supervision. The contribution of therapist involvement to reported effects cannot be fully disentangled from the effects of the technology itself.

## 12 Clinical Implications

The findings of this review support the use of technology as an adjunct to face‐to‐face SLT rather than a standalone intervention. CSLT demonstrated the most consistent evidence across intervention targets, particularly for word‐finding when delivered at sufficient dose and may extend practice beyond clinical contact hours for PWA capable of self‐directed engagement. VR showed promise for functional communication and language comprehension through immersive, socially meaningful practice contexts, while AI tools demonstrated potential for supporting aphasia assessment and classification, though neither is yet ready for standalone clinical use without further trial evidence. Across all technology types, adherence to the recommended therapy dose threshold was consistently associated with stronger outcomes, suggesting that the primary clinical value of technology lies in its capacity to make high‐intensity practice feasible and sustainable. For reading, writing and functional communication, the evidence remains insufficient for specific clinical recommendations, and larger, higher‐dose trials are needed.

## 13 Conclusion

This review identified three main types of technology used in aphasia assessment and rehabilitation: CSLT, VR and AI. AI has been used predominantly for assessment and diagnostic classification. In terms of intervention effectiveness, the strongest evidence relates to word‐finding, where studies delivering therapy at recommended doses show more consistent improvements. Studies achieving the recommended threshold of more than 20 h were more likely to report meaningful gains, though in many cases these gains may reflect the capacity of technology to enable greater practice intensity rather than the specific properties of the technology itself. For language production, comprehension, functional communication, reading and writing, technology‐supported interventions showed more mixed outcomes, which may reflect the influence of underdosed studies, varied study designs and small sample sizes. The certainty of evidence for these targets was generally moderate, except for writing, which received a low rating. Future research should examine technology‐supported interventions across all domains with larger samples and higher doses to fully understand their potential and may also explore whether integrating different types of technology could offer additional clinical benefit.

## Conflicts of Interest

Author Rebecca Palmer contributed to two studies reported across three papers in this review. This involvement did not influence the selection, appraisal, or interpretation of the evidence. No other competing interests are declared

## Supporting information




**Supporting File**: jlcd70322‐sup‐0001‐SuppMat.docx

## Data Availability

The data collection forms, extracted data from included studies and materials used for synthesis are available upon reasonable request from the corresponding author (First). This review applied a narrative synthesis approach for the results and did not involve statistical meta‐analysis.

## Appendix 1

### 1.1

Checklist items are worded so that one of five responses are possible (NICE 2023).

**Table:**

**++**	It shows that the study design has been structured to effectively reduce the potential for bias
**+**	It suggests that the response to the checklist question is either unclear from the study's reporting or the study may not have fully addressed all potential sources of bias.
−	Notable sources of bias are present.
Not reported (NR)	This should be used for aspects where the study under review does not provide information about them.
Not applicable (NA)	This should be applied to aspects of the study design that are not relevant to the study being reviewed.

## Appendix 2

### 2.1

The PRISMA 2020 checklist

**Table:**

Section and topic	Item #	Checklist item	Location where item is reported
**TITLE**			
Title	1	Identify the report as a systematic review.	Title page
**ABSTRACT**			
Abstract	2	See the PRISMA 2020 for Abstracts checklist.	Abstract
**INTRODUCTION**			1
Rationale	3	Describe the rationale for the review in the context of existing knowledge.	Background
Objectives	4	Provide an explicit statement of the objective(s) or question(s) the review addresses.	Abstract and Background
**Methods**			
Eligibility criteria	5	Specify the inclusion and exclusion criteria for the review and how studies were grouped for the syntheses.	Methods
Information sources	6	Specify all databases, registers, websites, organisations, reference lists and other sources searched or consulted to identify studies. Specify the date when each source was last searched or consulted	Methods
Search strategy	7	Specify the methods used to decide whether a study met the inclusion criteria of the review, including how many reviewers screened each record and each report retrieved, whether they worked independently, and if applicable, details of automation tools used in the process.	Methods
Selection process	8	Specify the methods used to collect data from reports, including how many reviewers collected data from each report, whether they worked independently, any processes for obtaining or confirming data from study investigators, and if applicable, details of automation tools used in the process.	Methods
Data collection process	9	Specify the methods used to collect data from reports, including how many reviewers collected data from each report, whether they worked independently, any processes for obtaining or confirming data from study investigators, and if applicable, details of automation tools used in the process.	Methods
Data items	10a	List and define all outcomes for which data were sought. Specify whether all results that were compatible with each outcome domain in each study were sought (e.g., for all measures, time points, analyses), and if not, the methods used to decide which results to collect.	Methods
	10b	List and define all other variables for which data were sought (e.g., participant and intervention characteristics, funding sources). Describe any assumptions made about any missing or unclear information.	Methods
Study risk of bias	11	Specify the methods used to assess risk of bias in the included studies, including details of the tool(s)used, how many reviewers assessed each study and whether they worked independently, and if applicable, details of automation tools used in the process.	Methods
Effect measures	12	Specify for each outcome the effect measure(s) (e.g., risk ratio, mean difference) used in the synthesis or presentation of results.	Methods
Synthesis methods	13a	Describe the processes used to decide which studies were eligible for each synthesis (e.g., tabulating the study intervention characteristics and comparing against the planned groups for each synthesis (item#5)).	Methods
	13b	Describe any methods required to prepare the data for presentation or synthesis, such as handling of missing summary statistics, or data conversions.	Methods
	13c	Describe any methods used to tabulate or visually display results of individual studies and syntheses.	Methods
	13d	Describe any methods used to synthesize results and provide a rationale for the choice(s). If meta‐analysis was performed, describe the model(s), method(s) to identify the presence and extent of statistical heterogeneity, and software package(s) used.	Methods
	13e	Describe any methods used to explore possible causes of heterogeneity among study results (e.g., subgroup analysis, meta‐regression).	Methods
	13f	Describe any sensitivity analyses conducted to assess robustness of the synthesized results.	Methods
Reporting bias assessment	14	Describe any methods used to assess risk of bias due to missing results in a synthesis (arising from reporting biases).	Methods
**Certainty assessment**	15	Describe any methods used to assess certainty (or confidence) in the body of evidence for an outcome.	Methods
**Results**			
Study selection	16a	Describe the results of the search and selection process, from the number of records identified in the search to the number of studies included in the review, ideally using a flow diagram.	Results
	16b	Cite studies that might appear to meet the inclusion criteria, but which were excluded, and explain why they were excluded.	Results
Study characteristics	17	Cite each included study and present its characteristics.	Results
Risk of bias in studies	18	Present assessments of risk of bias for each included study.	Results
Results of individual studies	19	For all outcomes, present, for each study: (a) summary statistics for each group (where appropriate) and (b) an effect estimates and its precision (e.g., confidence/credible interval), ideally using structured tables or plots.	Results
Results of syntheses	20a	For each synthesis, briefly summarise the characteristics and risk of bias among contributing studies.	Results
	20b	Present results of all statistical syntheses conducted. If meta‐analysis was done, present for each the summary estimate and its precision (e.g., confidence/credible interval) and measures of statistical heterogeneity. If comparing groups, describe the direction of the effect.	NA
	20c	Present results of all investigations of possible causes of heterogeneity among study results.	NA
	20d	Present results of all sensitivity analyses conducted to assess the robustness of the synthesized results.	NA
Reporting biases	21	Present assessments of risk of bias due to missing results (arising from reporting biases) for each synthesis assessed.	Results
Certainty of evidence	22	Present assessments of certainty (or confidence) in the body of evidence for each outcome assessed.	Results
**Discussion**	23a	Provide a general interpretation of the results in the context of other evidence.	Discussion
	23b	Discuss any limitations of the evidence included in the review.	Results & Discussion
	23c	Discuss any limitations of the review processes used.	Limitations of the current review
	23d	Discuss implications of the results for practice, policy, and future research.	Clinical implications
**OTHER INFORMATION**	24		
Registration and protocol	24a	Provide registration information for the review, including register name and registration number, or state that the review was not registered.	Abstract
	24b	Indicate where the review protocol can be accessed, or state that a protocol was not prepared.	Abstract & Methods
	24c	Describe and explain any amendments to information provided at registration or in the protocol.	Abstract & Methods
Support	25	Describe sources of financial or non‐financial support for the review, and the role of the funders or sponsors in the review.	NA
Competing interests	26	Declare any competing interests of review authors.	Competing interests
Availability of data, code and other materials	27	Report which of the following are publicly available and where they can be found template data collection forms; data extracted from included studies; data used for all analyses; analytic code; any other materials used in the review.	Availability of data, code and other materials

From: Page MJ, McKenzie JE, Bossuyt PM, Boutron I, Hoffmann TC, Mulrow CD, et al. The PRISMA 2020 statement: an updated guideline for reporting systematic reviews. BMJ 2021; 372: n71. https://doi.org/10.1136/bmj.n71. This work is licensed under CC BY 4.0. To view a copy of this license, visit https://creativecommons.org/licenses/by/4.0/.13 of 13.
