# Supplementary material for: Exploring the Effectiveness of Technology‐Based Interventions in Aphasia Rehabilitation: A Systematic Review
Source: Int J Lang Commun Disord. 2026 Sep 8;61(5):e70322. doi: 10.1111/1460-6984.70322 (PMC13551171; doi:10.1111/1460-6984.70322)
Supplement: Supplementary file 1 [file JLCD-61-0-s001.docx]

**Supplementary File 1: Search Strategy**

**Section A: Per-Database Search Strings**

***The same three-block search strategy was applied across all four databases, with one Scopus-specific adaptation (see notes). All searches were conducted in the title field only.***

| **Database** | **Platform** | **Search Block 1 — Population** | **Search Block 2 — Technology** | **Search Block 3 — Outcomes** | **Limits & Filters** |
| --- | --- | --- | --- | --- | --- |
| **PubMed** | NCBI PubMed | TITLE: (Aphasi* OR Dysphasi* OR PWA OR "Stroke survivors" OR "Stroke victims" OR "Stroke patients") | TITLE: ("Artificial intelligence" OR "Natural language processing" OR "Virtual Reality" OR "Augmented reality" OR Gamification OR Games OR Tech* OR "Tablet-based" OR Computer OR Software OR "Mobile technology" OR "Mobile apps") | TITLE: (Assessment OR Evaluation OR Intervention OR Treatment OR Therapy OR "self-manage*") | Language: English  Date (Phase 1): 2013–2024  Date (Phase 2): 2024–2026  Field: Title [TI]  Records retrieved:  Phase 1: part of ~1,159 total  Phase 2: 104 |
| **PsycINFO** | APA PsycINFO (via EBSCO or Ovid) | TITLE: (Aphasi* OR Dysphasi* OR PWA OR "Stroke survivors" OR "Stroke victims" OR "Stroke patients") | TITLE: ("Artificial intelligence" OR "Natural language processing" OR "Virtual Reality" OR "Augmented reality" OR Gamification OR Games OR Tech* OR "Tablet-based" OR Computer OR Software OR "Mobile technology" OR "Mobile apps") | TITLE: (Assessment OR Evaluation OR Intervention OR Treatment OR Therapy OR "self-manage*") | Language: English  Date (Phase 1): 2013–2024  Date (Phase 2): 2024–2026  Field: Title  Records retrieved:  Phase 1: part of ~1,159 total  Phase 2: 384 |
| **Web of Science** | Clarivate Web of Science Core Collection | TITLE: (Aphasi* OR Dysphasi* OR PWA OR "Stroke survivors" OR "Stroke victims" OR "Stroke patients") | TITLE: ("Artificial intelligence" OR "Natural language processing" OR "Virtual Reality" OR "Augmented reality" OR Gamification OR Games OR Tech* OR "Tablet-based" OR Computer OR Software OR "Mobile technology" OR "Mobile apps") | TITLE: (Assessment OR Evaluation OR Intervention OR Treatment OR Therapy OR "self-manage*") | Language: English  Date (Phase 1): 2013–2024  Date (Phase 2): 2024–2026  Field: Title  Records retrieved:  Phase 1: part of ~1,159 total  Phase 2: 405 |
| **Scopus** | Elsevier Scopus | TITLE: (Aphasi* OR Dysphasi* OR PWA OR "Stroke survivors" OR "Stroke victims" OR "Stroke patients") | TITLE: ("Artificial intelligence" OR "Natural language processing" OR "Virtual Reality" OR "Augmented reality" OR Gamification OR Games OR Technology OR Technologies OR "Tablet-based" OR Computer OR Software OR "Mobile technology" OR "Mobile apps") | TITLE: (Assessment OR Evaluation OR Intervention OR Treatment OR Therapy OR "self-manage*") | Language: English  Date (Phase 1): 2013–2024  Date (Phase 2): 2024–2026  Field: Title (TITLE field)  Note: Scopus does not support wildcard truncation (Tech*) inside complex multi-block title queries. Tech* was replaced with explicit terms: Technology; Technologies.  Records retrieved:  Phase 1: part of ~1,159 total  Phase 2: 266 |

**Section B: Search Notes and Additional Details**

| **Item** | Detail |
| --- | --- |
| **Search operator** | All three blocks connected by AND. Within each block, terms connected by OR. |
| **Field searched** | Title field only across all four databases. |
| **Language filter** | English only, applied in all four databases. |
| **Date range** | Phase 1 (original review): January 2013 to December 2024. Phase 2 (updated search, in response to reviewer concerns about search currency): January 2024 to June 2026. A one-year overlap (2024) was retained to ensure no eligible studies were missed at the boundary. |
| **Total records** | Phase 1: approximately 1,159 records across four databases before deduplication. Phase 2: 1,159 records (WoS: 405; PsycINFO: 384; Scopus: 266; PubMed: 104) before deduplication. |
| **Deduplication** | Duplicate records were identified and removed using Microsoft Excel prior to screening. |
| **Scopus adaptation** | Scopus does not support wildcard truncation (e.g. Tech*) within complex multi-block title field queries. The term Tech* was therefore replaced with the explicit terms Technology and Technologies in the Scopus search string only. All other terms were identical across databases. |
| **Grey literature & citation searching** | No grey literature search was conducted, and no backward or forward citation searching was performed. These are acknowledged as limitations in the main manuscript. |
